# Rac1 at the crossroad of actin dynamics and neuroinflammation in Amyotrophic Lateral Sclerosis

**DOI:** 10.3389/fncel.2014.00279

**Published:** 2014-09-08

**Authors:** Nadia D’Ambrosi, Simona Rossi, Valeria Gerbino, Mauro Cozzolino

**Affiliations:** ^1^Institute of Anatomy and Cell Biology, Università Cattolica del Sacro CuoreRome, Italy; ^2^National Research Council, Institute of Translational PharmacologyRome, Italy; ^3^Fondazione Santa Lucia IRCCSRome, Italy; ^4^Department of Biology, Università di Roma Tor VergataRome, Italy

**Keywords:** Amyotrophic Lateral Sclerosis (ALS), Rac1, neuroinflammation, motor neurons, NOX, microglia, reactive oxygen species, spinal muscular atrophy (SMA)

## Abstract

Rac1 is a major player of the Rho family of small GTPases that controls multiple cell signaling pathways, such as the organization of cytoskeleton (including adhesion and motility), cell proliferation, apoptosis and activation of immune cells. In the nervous system, in particular, Rac1 GTPase plays a key regulatory function of both actin and microtubule cytoskeletal dynamics and thus it is central to axonal growth and stability, as well as dendrite and spine structural plasticity. Rac1 is also a crucial regulator of NADPH-dependent membrane oxidase (NOX), a prominent source of reactive oxygen species (ROS), thus having a central role in the inflammatory response and neurotoxicity mediated by microglia cells in the nervous system. As such, alterations in Rac1 activity might well be involved in the processes that give rise to Amyotrophic Lateral Sclerosis (ALS), a complex syndrome where cytoskeletal disturbances in motor neurons and redox alterations in the inflammatory compartment play pivotal and synergic roles in the final disease outcomes. Here we will discuss the genetic and mechanistic evidence indicating the relevance of Rac1 dysregulation in the pathogenesis of ALS.

## Introduction

Degeneration of motor neurons in the brain cortex, brainstem and spinal cord of patients is the most recognized feature of Amyotrophic Lateral Sclerosis (ALS). Since the discovery of the first gene associated to familial forms of ALS, *Sod1*, many fundamental achievements in clinical, genetic, and basic biology processes related to ALS have profoundly improved our knowledge of the disease (Cozzolino et al., 2012). Indeed, while it has long been thinking that ALS was a disease highly specific for motor neurons, with a low albeit significant incidence of genetics limited to familial forms, it is now widely accepted that ALS is a complex syndrome that involves a wide range of different tissues and cell types, including muscle cells, astrocytes, microglia, oligodendrocytes. Further, it is clear that genes have a profound impact on the overall disease pathogenesis, even in apparently sporadic forms (Renton et al., 2014). To make things more intricate, many of the recently identified gene mutations that are definitely involved in familial (and to some degrees in sporadic) forms of ALS are also responsible of, or associated to diseases that do not primarily manifest as motor neuron diseases, ranging from Fronto Temporal Dementia (FTD) to Huntington Disease Phenocopies, putting in the spotlight the concept that ALS is part of a more widespread and complex disease process (Cooper-Knock et al., 2014).

Although different mechanisms have been invoked to play a role in the pathogenesis of ALS (Cozzolino et al., 2012), RNA metabolism has emerged as a fundamental aspect of disease determination. Two different lines of reason support this conclusion. First, recent genetic evidence pinpointed at least three different genes whose mutations, together accounting for nearly 50% of familial ALS cases, are likely responsible for disease-relevant alterations in RNA control. These genes include *TARDBP* (Neumann et al., 2006), *FUS/TLS* (Vance et al., 2009), and *c9orf72* (DeJesus-Hernandez et al., 2011; Renton et al., 2011). In all cases, mutations in these genes are believed to alter the control of RNA processing, including mRNA transcription, splicing, translation and transport (Ling et al., 2013), although the particular step of RNA metabolism that is affected in ALS is still to be ascertained.

Second, analogies with another Motor Neuron Disease (MND), namely Spinal Muscular Atrophy (SMA), sustain the idea that post-transcriptional gene regulation is profoundly affected in MND, including ALS. Indeed, SMA is caused by a decreased availability of the Survival Motor Neuron protein (SMN), due to gene mutations or deletions in the *smn1* gene, and this leads to an early degeneration of lower motor neurons in children. SMN is known to play a crucial function both in the biogenesis and localization of small ribonucleoprotein particles (snRNPs), the core modules of the splicing machinery (Burghes and Beattie, 2009), and in the transport along neuronal processes of cytosolic mRNAs-containing granules (Rage et al., 2013), an essential process that guarantees local protein synthesis (Jung et al., 2014). This is particularly important in, although not limited to, neurons, where local mRNA translation sustains axon elongation and maintenance, as well as synapses formation and plasticity (Jung et al., 2012). Thus, in the complete absence of SMN, cells and animals die (Schrank et al., 1997), to testify the widespread and essential role of SMN, while the shortage of SMN protein is particularly harmful to neurons. Although the reasons why a decreased availability of SMN activates a very selective process that leads to the specific degeneration of lower motor neurons is far from being clear, evidence exist that mys-functioning in SMN-regulated processes might be shared by ALS (Yamazaki et al., 2012; Gerbino et al., 2013; Groen et al., 2013; Turner et al., 2014), further indicating that motor neurons are particularly vulnerable to disturbed RNA metabolism.

## Evidence pointing to dys-regulation of actin dynamics in MNDs: Rho GTPases and motor neurons

The overlap between ALS and SMA is not however restricted to RNA control. Indeed, ALS and SMA share actin dynamics disturbances as a central route for motor neuron degeneration. In particular, the regulation of actin dynamics at the synapse, which is accomplished by a plethora of actin-binding/-regulating proteins is crucial for the proper function and maturation of neuromuscular junctions (NMJs), that are supposed to be a primary, early target of both ALS and SMA (Fischer et al., 2004; Kariya et al., 2008).

Both β-actin mRNA and protein are significantly reduced in distal axons and growth cones of SMA motor neurons, thus establishing a link between actin perturbations to the degeneration of NMJs in SMA (Rossoll et al., 2003). Similarly, overexpression of SMN in cultured neuronal cells promotes neurite outgrowth, while downregulation has an opposite effect (van Bergeijk et al., 2007). This is the consequence of changes in the ratio between F- and G-actin, further indicating that SMN regulates actin dynamics. Actually, the overexpression in SMA motor neurons of the actin-binding protein plastin 3, a genetic modifier for SMA (Oprea et al., 2008), rescues defects due to decreased SMN (Ackermann et al., 2013). Microfilament dynamics are also apparently mediated by profilins (Birbach, 2008) and SMN was shown, in particular, to bind and regulate the expression of profilin2a, which is known to promote actin polymerization (Nölle et al., 2011). Profilin2a is a key player in the Rho-kinase (ROCK) pathway, and loss of SMN results in an increased activity of the small GTPase RhoA, and an increased binding of profilin2a to ROCK, a direct downstream effector of RhoA. RhoA-mediated activation of ROCK then leads to a consequent ROCK-dependent hyper-phosphorylation of profilin2a which in this form eventually represses neurite outgrowth in neuronal cells (Bowerman et al., 2010; Nölle et al., 2011). The functional relevance of this pathway is clearly demonstrated by the observation that treating SMA mice with ROCK inhibitors strongly improves the maturation of the NMJs, increases muscle fiber size and prolongs their survival (Bowerman et al., 2010, 2012).

Motor neuron loss is also delayed in experimental models of ALS by fasudil, a clinical approved ROCK inhibitor (Takata et al., 2013). These results thus indicate that a similar pattern of deregulation affecting the RhoA/ROCK/profilin pathway might be operating in the degenerating motor neurons in ALS. Mutations within the *profilin 1* (PFN1) gene have been identified in late 2012 to cause ALS in two large families (Wu et al., 2012). PFN1 is crucial for the conversion of monomeric to filamentous actin, and ALS-linked PFN1 mutations were shown to reduce the ability of PFN1 to bind actin and to induce a decrease in the ratio of F/G actin. This eventually leads to inhibition of neurite outgrowth and alteration in growth cone morphology, all features clearly reminiscent of actin dynamics disturbances. Although later mutational screening in worldwide ALS populations established that PFN1 gene mutations are a very rare cause of familial ALS (van Blitterswijk et al., 2013), these findings, together with the association of familial ALS to rare mutation in cytoskeleton-regulating genes such as neurofilament heavy polypeptide, peripherin and dynactin provide a considerable genetic basis to disruption of actin cytoskeleton as a possible pathogenic mechanism in motor neuron degeneration (Renton et al., 2014).

As central regulators of actin dynamics, Rho family of small GTPases, with RhoA, Rac1 and cdc42 the most studied members, are natural candidates to play a role in these processes. A growing body of evidence suggests, in particular, that inhibition of Rac1 mediates the process of motor neuron degeneration in ALS. Mutations in *ALS2* were described to be responsible for a recessive form of juvenile-onset ALS (Yang et al., 2001), although later studies have suggested that *ALS2*-induced disease phenotypes are more related to slowly progressive, juvenile forms of hereditary spastic paraplegia (Panzeri et al., 2006). Nonetheless, *ALS2* encodes for the protein alsin, which has been characterized as a guanine nucleotide exchange factor (GEF) for Rho family of small GTPases, including Rac1, and mutation in alsin are expected to affect GEF activity of this protein (Yang et al., 2001; Topp et al., 2004). In line with its activatory function, alsin knockdown inhibits axon growth and induces cell death in cultured motor neurons, further indicating the importance of cytoskeletal balance in motor neuron health. Importantly, cell death and cytoskeletal alterations are obtained in the same cells by overexpression of a dominant-negative Rac1 mutant, while a constitutively active Rac1 mutant is able to inhibit it (Jacquier et al., 2006). Moreover, the apoptotic phenotypes induced in NSC34 motor neuron-like cells by overexpression of mutSOD1 are inhibited by co-expression of alsin, and this neuroprotective activity is completely abolished by decreasing endogenous Rac1 with siRNA (Kanekura et al., 2005), indicating that Rac1 acts downstream of alsin. Overall, these observations clearly indicate that disruption of Rac1 GTPase function is a contributing event in motor neuron degeneration in ALS. This conclusion is further supported by findings from experiments in cultured neuronal cells depleted of TDP43. Indeed, TDP43 knockdown in neuronal cells inhibits neurite outgrowth and induces cell death, as a consequence of decreased membrane association and inactivation of RhoA, Rac1, and cdc42 Rho GTPases (Iguchi et al., 2009). However, it has also been shown that TDP43 knockdown in hippocampal neurons is able to repress Rac1 expression at a translational level, and this causes a significant increase in the density of dendritic spines (Majumder et al., 2012), thus further indicating that the outcome of Rho GTPases activity is indeed a unique feature depending on the specific neuronal cell subpopulation.

All these results point to Rac1-dependent cytoskeletal dys-regulation as a key determinant of motor neuron degeneration. However, beyond their characterized function in actin dynamics, Rho GTPase signaling is also involved in other important cellular processes (Linseman and Loucks, 2008) that might be altered in ALS. An important link between Rac1 and motoneuronal cell viability was provided by the observation that the overexpression of mutSOD1 proteins decreases the levels of active, GTP-bound form of Rac1 and induces apoptosis in SH-SY5Y neuronal cell lines (Pesaresi et al., 2011). Importantly, the overexpression of a constitutive active form of Rac1 (V12) fully protects SH-SY5Y cells from apoptosis, whereas a dominant-negative, inactive form of mutant of Rac1 (N17) is *per se* sufficient to induce apoptotic cell death. Thus, Rac1 inhibition is anticipated to have a critical role in motor neurons demise in ALS. Yet, a direct evidence that this is occurring in motor neurons *in vivo*, has not been reported so far. Interestingly, the inhibition of Rac1 by mutSOD1s is strictly dependent on the activation of a signaling pathway which originates from mitochondria and is mediated by the p66KDa isoform of the adaptor protein Shc (p66Shc): when mitochondrial damage is prevented by p66Shc functional inhibition, Rac1 activity is rescued, and cell survival recovered. Thus, damage to mitochondria, which represent a well recognized, early sign of motor neuron degeneration in ALS, might represent the trigger of a pathological cascade involving Rac1 dysregulation, eventually leading to both actin cytoskeleton disturbances and loss of cell viability. In addition, these data point to mitochondria-derived reactive oxygen species (ROS) as mediators of Rac1 inhibition. Many types of hints are indeed accumulating, which indicate that Rho family GTPases are a redox sensitive family of proteins (Mitchell et al., 2013). This is of crucial importance in ALS, a pathological condition that is associated to critical alterations in the redox balance which exert detrimental effects not only at the level of NMJ (Pollari et al., 2014), but also in the inflammatory compartment where ROS-mediated microglial cells activation plays a fundamental role in ALS disease progression (Boillée et al., 2006).

## Rac1 as a major player in inflammation in ALS

The neuroinflammatory process is a feature common to all ALS forms, either sporadic or familial, and in the latter case all ALS-causative genes exert a comparable activation of the glial compartment, despite their specific function (Philips and Robberecht, 2011; Rizzo et al., 2014). It is commonly believed that the neuroinflammatory reaction played in particular by microglia, the resident immune cells of the CNS, exerts a protective function at early stages of the pathology that is switched into a detrimental one as the disease progresses (Appel et al., 2011). Accordingly, microglia cells producing trophic, beneficial effects are said to belong to the M2 phenotype, whilst the toxic phenotype is defined as M1. In response to stimuli derived from damaged motor neurons and from autocrine/paracrine glial signaling, M1 microglia produces and releases ROS and pro-inflammatory cytokines that are responsible for a self-propagating cycle of progressive and irreversible motor neuron death (Henkel et al., 2009). The inhibition of such damaging pro-inflammatory process is therefore a main field in ALS research and one target of interest in this context is NOX.

In particular, NOX2 (gp91phox) is a major source of superoxide by phagocytic cells (Bedard and Krause, 2007), and is the predominant isoform found in microglia, astrocytes and neurons (Sorce and Krause, 2009), where its activation results in a neurotoxic outcome (Marchetto et al., 2008; Liao et al., 2012). NOX2 expression is upregulated both in familial and sporadic ALS patients and in different ALS-animal models, and NOX2 inhibition by genetic ablation in SOD1-G93A mice significantly slows disease progression and extends survival (Wu et al., 2006; Marden et al., 2007). Pharmacological inhibition of NOX2 by apocynin was shown to protect motor neurons from mutant SOD1 toxicity in culture (Harraz et al., 2008; Marchetto et al., 2008; Trumbull et al., 2012), but gave contrasting results when administered to SOD1-G93A mice (Harraz et al., 2008; Trumbull et al., 2012). Anyway, NOX2 is considered one of the main markers of M1-toxic microglia. NOX2 consists of the integral membrane subunits gp91^Phox^ and p22^Phox^, which are permanently associated with each other to form the catalytic flavocytochrome *b*_558_ core (cyt *b*_558_), the cytosolic subunits p40^Phox^, p47^Phox^, p67^Phox^ and Rac1. Upon activation, the cytosolic factors translocate to membranes to interact with cytb_558_, through which electrons from NADPH are transported to reduce molecular oxygen to superoxide. Active, GTP-bound Rac1 acts both as an adaptor, to ensure correct positioning of p67^Phox^ toward cytb_558_, and as a player in the electron-transfer reaction (Bedard and Krause, 2007).

In ALS, uncontrolled Rac1 activation represents a straight link between disease-causing mutSOD1s and NOX2-mediated ROS production by glial cells. It was in fact clearly demonstrated that SOD1 directly binds to Rac1 in a redox-sensitive manner, influencing the rate of intrinsic GTP hydrolysis, and thus of Rac1 activity: in reducing conditions SOD1 binds to Rac1 and stimulates its activity; conversely, in oxidizing conditions SOD1 dissociates from Rac1, favoring GTP to GDP exchange and thus eventually silencing Rac1-dependent activity of NOX2. This mechanism is found dysregulated in glial cells by different SOD1 mutants, that are less sensitive to redox uncoupling, in particular in a subpopulation of early endosomes containing NOX2, called redoxosomes (Harraz et al., 2008; Li et al., 2011). As a matter of fact, expression of mutSOD1s in glial cells elevates the levels of GTP-bound Rac1 (Harraz et al., 2008), that are also found dramatically increased in microglia derived from SOD1-G93A mice (Apolloni et al., 2013). ROS generated by the Rac1-NOX2 axis are then responsible for TNFα secretion, autocrine and paracrine induction of the pro-inflammatory transcription factor NFκB in co-cultured neurons and neurotoxicity (Li et al., 2011). Substantial raise of active Rac1 is also found in spinal cord from SOD1-G93A mice, with consequent hyperactivation of NOX2 (Harraz et al., 2008).

The overactivation of Rac1/NOX2 pathway *in vivo* could be also the resultant of the action of different pro-inflammatory molecules, such as extracellular ATP. This nucleotide is an activator of microglia response through purinergic receptors and it is supposed to be increasingly released during neuronal damage. In ALS, the P2X7 receptor preferential agonist 2′-3′-*O*-(benzoyl-benzoyl) ATP, enhances the neurotoxic potential of SOD1-G93A microglia and increases the production of ROS through the recruitment of Rac1 and NOX2. Conversely, different P2X7 antagonists restore ROS production to basal levels decreasing the amount of Rac1-GTP and inhibiting NOX2 activity (Apolloni et al., 2013).

A role for Rac1 in ALS neuroinflammation is also suggested by the interplay between alsin and SOD1-G93A in influencing the dynamics of ROS production in glial cells. Alsin is known to bind three components of the redoxosome: it possesses Rho-GEF domains for Rac1 and Rab5 and it also interacts with SOD1. When coexpressed with SOD1-G93A, alsin was shown to attenuate SOD1-dependent Rac1 activation, ROS generation by NOX2, NFκB induction, TNFα secretion and to protect neurons from toxicity in co-culture studies (Li et al., 2011). This protective effect is in line with the detrimental one exerted by alsin knockdown in motor neurons (Jacquier et al., 2006) and appears to be mediated by the ability of alsin to decrease Rac1 activation in the presence of SOD1-G93A. These findings suggest that independent ALS disease-associated genes may converge on the same pathogenic pathway, i.e., the Rac1/NOX2 ROS-generating mechanism.

## Therapeutic perspectives targeting Rac1

In this review, we have shown how cytoskeletal dynamics alterations in motor neurons as well as neuroinflammatory events in microglia underlie the pathogenesis of ALS and other MNDs, and how Rac1 GTPase might be involved in both processes. Although many gaps exist in our knowledge about the mechanistic aspects of these processes, Rac1 should be considered as a valuable target to interfere with the disease development.

Like other Rho GTPases, Rac1 represents a hub that responds to different signaling networks and in turn results in different, even contrasting outcomes by interacting with many diverse downstream effectors, that might well be different depending on the cell type as well as the stimulus involved. Thus, the apparently contradictory evidence about the activity of Rac1 in ALS motor neurons and microglia should not be regarded as surprising. Yet, the antagonistic effects that Rac1 targeting might have on the motor neurons as opposed to the inflammatory compartments poses some questions about how to devise an effective strategy, that will need to be answered in the near future. Nonetheless, a significant body of studies performed in contexts where aberrant Rac1 signaling is associated to alterations in neuronal structural plasticity and actin cytoskeleton dynamics indicates that the modulation of Rac1-dependent signaling pathways could be effective to protect neuronal degeneration (Lorenzetto et al., 2013; Tönges et al., 2014; Travaglione et al., 2014). Of course, an accurate preclinical evaluation in animal models of ALS, that are widely available, will help answering these questions.

## Conflict of interest statement

The authors declare that the research was conducted in the absence of any commercial or financial relationships that could be construed as a potential conflict of interest.
